# Nutritional Omega-3 Deficiency Alters Glucocorticoid Receptor-Signaling Pathway and Neuronal Morphology in Regionally Distinct Brain Structures Associated with Emotional Deficits

**DOI:** 10.1155/2016/8574830

**Published:** 2015-12-30

**Authors:** Thomas Larrieu, Muna L. Hilal, Véronique De Smedt-Peyrusse, Nathalie Sans, Sophie Layé

**Affiliations:** ^1^INRA, Nutrition et Neurobiologie Intégrée, UMR 1286, 33076 Bordeaux, France; ^2^Univ. Bordeaux, Nutrition et Neurobiologie Intégrée, UMR 1286, 33076 Bordeaux, France; ^3^Brain Mind Institute, School of Life Sciences, École Polytechnique Federale de Lausanne (EPFL), 1050 Lausanne, Switzerland; ^4^INSERM, Neurocentre Magendie, UMR 862, 33077 Bordeaux, France; ^5^Univ. Bordeaux, Neurocentre Magendie, UMR 862, 33077 Bordeaux, France

## Abstract

Extensive evidence suggests that long term dietary n-3 polyunsaturated fatty acids (PUFAs) deficiency results in altered emotional behaviour. We have recently demonstrated that n-3 PUFAs deficiency induces emotional alterations through abnormal corticosterone secretion which leads to altered dendritic arborisation in the prefrontal cortex (PFC). Here we show that hypothalamic-pituitary-adrenal (HPA) axis feedback inhibition was not compromised in n-3 deficient mice. Rather, glucocorticoid receptor (GR) signaling pathway was inactivated in the PFC but not in the hippocampus of n-3 deficient mice. Consequently, only dendritic arborisation in PFC was affected by dietary n-3 PUFAs deficiency. In addition, occlusion experiment with GR blockade altered GR signaling in the PFC of control mice, with no further alterations in n-3 deficient mice. In conclusion, n-3 PUFAs deficiency compromised PFC, leading to dendritic atrophy, but did not change hippocampal GR function and dendritic arborisation. We argue that this GR sensitivity contributes to n-3 PUFAs deficiency-related emotional behaviour deficits.

## 1. Introduction

Early life stress, including malnutrition, results in altered synaptic and behavioural functions in adult life [1–3]. Among the myriad of components of food, polyunsaturated fatty acids (PUFAs) have received substantial consideration as being relevant to many diseases, including anxiety and depression [4–6]. A compelling body of evidence reveals that anxiety and depressive disorders are linked to dietary lipids, especially the n-3 PUFAs [1, 7–12]. Dysfunction of the hypothalamic-pituitary adrenal (HPA) axis including glucocorticoid receptor (GR) signaling pathway remains one of the cardinal features of depression and anxiety [13–17]. Although several mechanisms underlying the effects of dietary n-3 PUFAs deficiency on emotional behaviour have been described (see [18] for review), those specifically related to HPA axis function remain poorly understood. Using an animal model of maternal dietary n-3 PUFAs deficiency, we recently found that mice that were fed a deficient diet in n-3 PUFAs were under a chronic stress state reflected by behavioural and neuronal changes that resemble those of mice exposed to social defeat stress. These effects were mediated by HPA axis hyperactivity and were reversed by n-3 PUFAs supplementation [19]. Despite the clear importance of dietary n-3 PUFAs in maintaining HPA axis function and preventing emotional impairment, mechanisms by which n-3 PUFAs deficiency induces HPA axis hyperactivity remain largely unexplored. Here, we confirmed and followed up on our initial observations by determining of the processes by which nutritional n-3 PUFAs deficiency induces HPA axis hyperactivity. To do so, we aimed at investigating the effects of maternal dietary n-3 PUFAs deficiency on GR-mediated HPA axis feedback inhibition along with GR signaling pathway and neuronal arborisation in prefrontal and hippocampal brain structures. Here we found that GR signaling pathway was inactivated in the prefrontal cortex (PFC) but not in the hippocampus of omega-3 deficient mice. Consequently, only dendritic arborisation in PFC was affected by dietary n-3 PUFAs deficiency. In addition, occlusion experiment with GR blockade altered GR signaling in the PFC of control mice as well as anxiety and social behaviour with no further alterations in n-3 deficient mice. We argue that this GR sensitivity contributes to n-3 PUFAs deficiency-related HPA axis deregulation.

## 2. Methods 

### 2.1. Animals

All experiments were performed according to criteria of the European Communities Council Directive (50120103-A). Behavioural and biochemical experiments were performed on C57Bl6/J mice obtained from Charles River (L'Arbresle, France). Mice were maintained under standard housing conditions on corn cob litter in a temperature-controlled (23 ± 1°C) and humidity-controlled (40%) animal room with a 12 h light/dark cycle (7:00–19:00), with ad libitum access to food and water. CD1 mice used as the social target were obtained from Charles River. All tests were conducted during the light period. C57BL6/J male mice were housed individually and were 3-4 months old when the behavioural analysis and biochemical analysis were conducted.

### 2.2. Diets

C57Bl6/J mice were given water and isocaloric experimental diets ad libitum (pellets prepared by UPAE-INRA, Jouy-en-Josas, France, replaced daily) as previously described [1, 20, 21]. After mating, C57BL6/J females were fed throughout gestation and lactation with a diet containing 6% of rapeseed oil (rich in *α*-linolenic acid, 18:3n-3; the control diet) or 6% fat in the form of sunflower oil (rich in linoleic acid, 18:2n-6; the n-3 deficient diet). After weaning, male offspring were fed with the same diet as their dam until the end of the experiments.

### 2.3. Surgery and Mifepristone Treatment

For pellets implantation, the skin was lifted on the back side of the C57BL6/J male mice and an incision was made. A pocket was formed with a pair of forceps about 2 cm beyond the incision site. Finally, mifepristone or placebo pellets were implanted into the pocket with forceps. Mifepristone pellets released continuously 20 mg/kg/day for 21 days (Innovative Research of America (IRA)). Innocuousness of the compound at this dose and time has been revealed by measuring body weight change and locomotor activity (data not shown). After 21 days of treatment, open-field and social interaction tests were performed as described below. (see Figure 3(a) for the timeline).

### 2.4. Behavioural Testing

Social interaction measurement was performed as previously published [1, 20]. Mice were transferred to a new cage (40 × 40 cm). A social interaction session comprised 5 minutes without target followed by 5-minute exposure of an unfamiliar adult CD1 male enclosed in a wire mesh placed in the corner of the field. Number of active investigatory behaviours (mainly sniffing the anogenital region, mouth, ears, trunk, and tail) was manually counted by an experimenter blind to the conditions.

Open-field test was performed as previously published [19, 20]. The apparatus consisted of a Plexiglas open-field (40 × 40 cm) with 16 cm high walls. Lighting consisted of four fluorescent bulbs at a height of 2 m above the floor of an open-field apparatus placed on each corner of the experiment room (light intensity of 35 Lux). The floor was cleaned between each trial to avoid olfactory clues. Each mouse was transferred to an open field facing a corner and was allowed to freely explore for 10 minutes an open field. A video tracking system (Smart, Panlab, Spain) recorded the exact track of each mouse as well as total distance travelled (cm) and the percentage of time spent in the centre.

### 2.5. Plasmatic Corticosterone Analysis

Trunk blood collection in ethylenediaminetetraacetic acid-lined tubes (EDTA) was performed during diurnal rise period, previously determined to occur 60 minutes before lights went off. Corticosterone was measured with an in-house RIA in the plasma as previously described [19]. Briefly, after steroid extraction with absolute ethanol, total corticosterone was measured by competition between cold corticosterone (B) and 3H-B (B^*∗*^) by a specific anti-corticosterone antibody provided by Dr. H. Vaudry (University of Rouen, France).

### 2.6. Morphological Analysis

Brains were quickly removed, washed in PBS, and processed for staining of individual neurons following the manufacturer's instructions for the rapid Golgi kit (FD Neurotech). Golgi stained brain slices of 100 *μ*m containing the PFC were used for morphological analysis. Pyramidal neurons of dorsal CA1 region of hippocampus (from −1.34 to −2.30 mm anterior to bregma) and PFC II/III layers were chosen for morphological analysis in our study. The dorsal PFC can be divided into dorsolateral PFC including frontal association (FrA) cortex (from 2.58 to 3.08 mm anterior to bregma) and dorsomedial PFC including prelimbic (PL) cortex (from 1.5 to 2.3 mm anterior to bregma). Pyramidal neurons within this region are defined as having a cell body which is immediately lateral to layer I, which is relatively absent of cells. These neurons are also defined by the presence of a basilar dendritic tree and a clearly defined single apical dendrite that projects toward the pial surface. For analysis, cells that met the following criteria were chosen: (1) relative isolation of the cell body from neighbouring impregnated neurons; (2) cell bodies existing between 150 and 250 *μ*m from the pial surface to prevent artefacts due to unrepresentative sampling from neurons of varying distance from the midline; and (3) the presence of intact primary, secondary, and tertiary dendrites. As our previous study, which has examined the effects of nutritional omega-3 deficiency and stress on this neuronal class within the mPFC, has found selective effects on the apical dendrites, as opposed to the basal dendrites, analysis was restricted to the apical dendritic tree. Three to 5 neurons per mouse and per region were reconstructed by a trained experimenter blind to the conditions using a Zeiss microscope Axio Imager 2 (×100) and analysed using the Neurolucida software. Sholl analysis was performed, in which the centre of the soma was used as a reference point and dendritic length was quantified both as a total measure per cell and as a function of radial distance from the soma in 10 *μ*m increments.

### 2.7. Western Blot Analysis

One day after the last behavioural test, mice used for the occlusion experiment were sacrificed and the brain was quickly removed from the skull and rinsed in cold Milli-Q water to remove any surface blood. Prefrontal cortex was collected with a blade and forceps after removing the olfactory bulbs. Right after rolling out the cortex from both hemispheres, hippocampus was dissected from the cortex using forceps. Collected tissues were flash frozen in dry ice and stored at −80°C until analysis. Western blot measurement was performed as previously described [19, 20]. PFC and hippocampus were homogenised in lysis buffer (TRIS 20 mM pH 7.5, antiprotease cocktail, 5 mM MgCl2, 1 mM DTT, 0.5 M EDTA, 1 mM NaOV, and 1 mM NaF). After centrifugation, protein concentration was determined using a BCA assay kit (Uptima, Montluçon, France). Equal amounts of proteins (50 *μ*g) were loaded onto SDS-PAGE gel (10%) and transferred onto PVDF membrane (Millipore, Billerica, MA, USA). Membranes were incubated overnight (4°C) with anti-GR (M-20) (1 : 5000, Santa Cruz Biotechnology, Santa Cruz, CA, USA), anti-FKBP51 (1 : 500, Santa Cruz Biotechnology), anti-E6AP (H-182) (1 : 500, Santa Cruz Biotechnology), and anti-actin (1 : 2500, Sigma, Saint-Louis, MS, USA) antibodies. After washing, membranes were incubated 1 h with rabbit peroxidase-conjugated secondary antibody (1 : 5000, Jackson ImmunoResearch laboratories, West Grove). Between each revelation, membranes were incubated 15 minutes in stripping buffer (Re-Blot Plus, Millipore) to remove the previous antibody. Staining was revealed with ECL-Plus Western blotting system (Perkin Elmer, Forest City, CA). Chemiluminescence was captured and quantified using GeneTools software (Syngene).

### 2.8. Dexamethasone Suppression Test

The mice received a single intraperitoneal injection of dexamethasone dissolved in saline (0.9% NaCl, 0.1 mg per kg; 0.1 mL per 10 g of mouse; Sigma-Aldrich, France) or 0.9% NaCl in control condition 6 hours before decapitation and determination of total corticosterone levels in plasma as previously described [22].

### 2.9. Statistical Analyses

All values are given as mean ± SEM. Results obtained in mifepristone and dexamethasone experiments were all analysed by a two-way analysis of variance (ANOVA), with treatment and diet as fixed factors. Analyses were followed by Bonferroni post hoc test when appropriate. Results obtained in morphological experiments were all analysed by an unpaired *t*-test. All statistical tests were performed with GraphPad Prism (GraphPad software) using a critical probability of *p* < 0.05. Statistical analyses performed for each experiment are summarised in each legend of figures with the chosen statistical test, *n* and *p* values, degree of freedom, and *F*/*t* values.

## 3. Results

**Figure 1:**
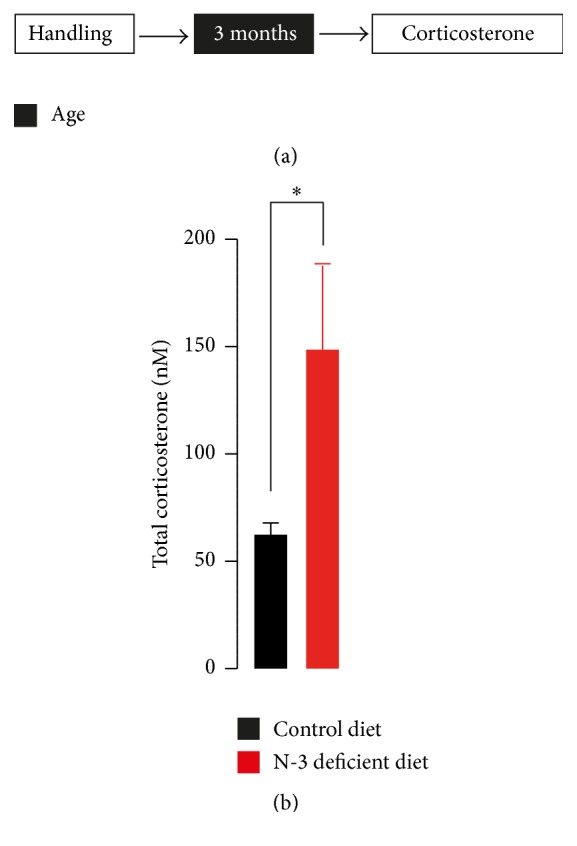
Effects of n-3 deficient diet on total plasma corticosterone levels. N-3 deficient mice showed total corticosterone elevation as compared to control diet mice in steady-state condition (*t*_7_ = 2.472, ^*∗*^ *p* < 0.05, unpaired *t*-test, *n* = 4-5 per group). Data are displayed as mean ± SEM.

**Figure 2:**
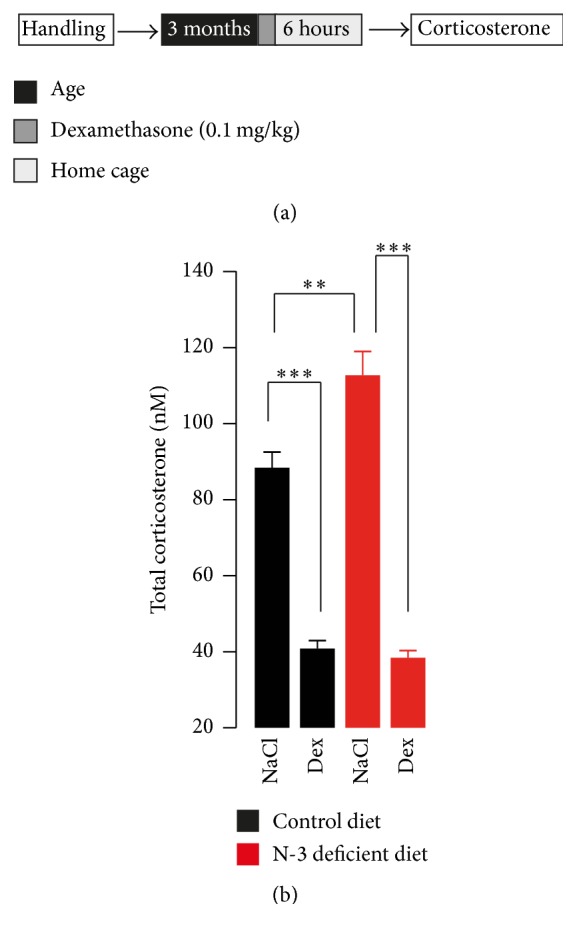
Corticosterone levels after dexamethasone application. (a) Experimental timeline. The mice received a single intraperitoneal injection of dexamethasone dissolved in saline or NaCl 6 hours before decapitation and determination of total corticosterone levels in plasma. (b) A significant interaction between diet and treatment was revealed on corticosterone suppression (interaction: *F*_1,22_ = 12.58, *p* < 0.01, two-way ANOVA, Bonferroni's test; *n* = 6-7 per group). After administration of dexamethasone both control diet and n-3 deficient mice showed decreased corticosterone levels in plasma. Data are displayed as mean ± SEM.

We investigated plasma corticosterone levels in undisturbed control diet and n-3 deficient mice to confirm our previous data [19].  Figure 1 shows that dietary n-3 PUFAs deficiency induced a significant increase in total plasma corticosterone levels compared with control diet in basal conditions. In order to understand how dietary n-3 PUFAs deficiency leads to corticosterone hypersecretion, we then tested whether HPA axis feedback regulation was compromised in n-3 deficient mice. Some depressed patients and mice that display depression-related behaviours show impaired suppression of endogenous glucocorticoids by the synthetic glucocorticoid dexamethasone [23]. For this purpose, we used this clinically established neuroendocrinological test, the dexamethasone suppression test [22]. We found that dexamethasone at the dose of 0.1 mg/kg effectively suppressed the corticosterone release in control diet mice (Figure 2). Surprisingly, we found that, six hours after dexamethasone treatment, n-3 deficient mice also exhibited a decrease in total corticosterone levels in plasma as compared to their respective saline group. This result suggests that HPA axis feedback induced by dexamethasone is intact in mice fed with a deficient diet in n-3 PUFAs.

**Figure 3:**
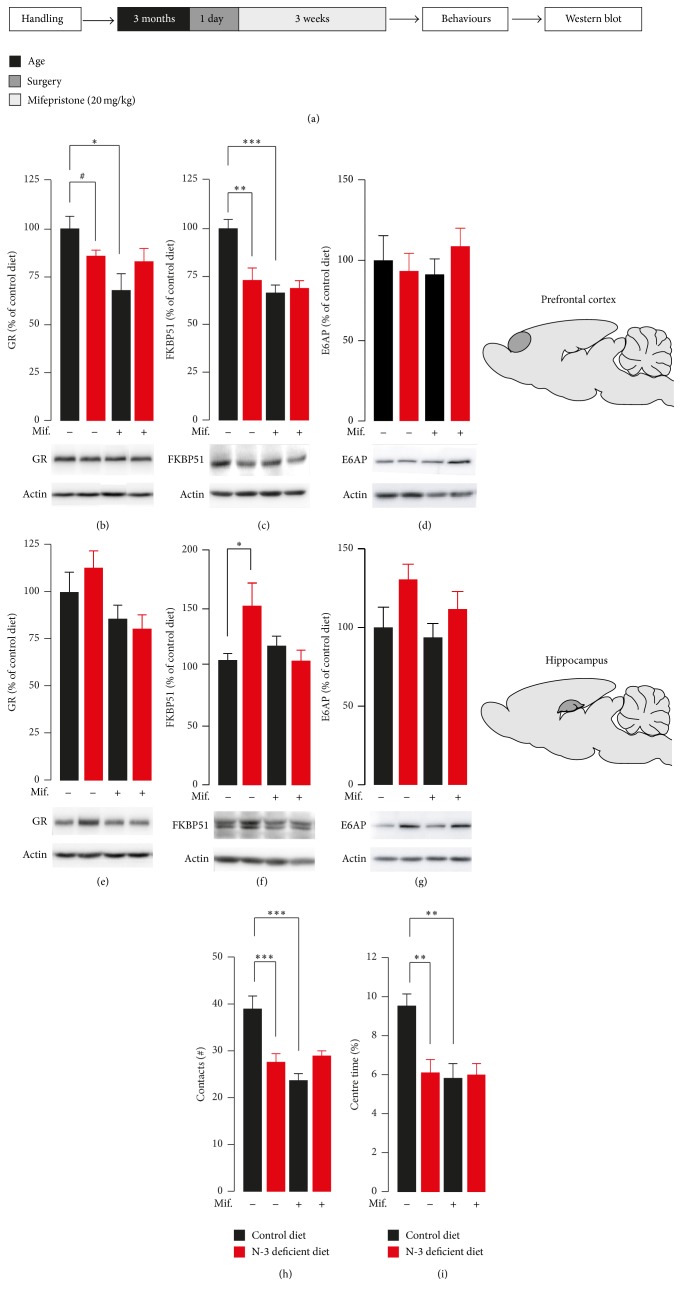
Effects of n-3 deficient diet on GR signaling pathway and emotional behaviours. (a) Experimental timeline. Mifepristone pellets released continuously 20 mg/kg/day for 21 days. After 21 days of treatment, open-field and social interaction tests were performed. One day after the last behavioural test, mice were sacrificed for Western blot analysis. (b, c) A significant interaction between diet and treatment was revealed on both (b) GR (interaction: *F*_1,14_ = 5.540, *p* < 0.05, two-way ANOVA, Bonferroni's test; *n* = 4-5 per group) and (c) FKBP51 expression in the PFC (interaction: *F*_1,17_ = 7.584, *p* < 0.05, two-way ANOVA, Bonferroni's test; *n* = 4-5 per group). (d) No change was revealed in the expression of E6AP in the PFC of mice (interaction: *F*_1,21_ = 0.9281, *p* = 0.3463, diet effect: *F*_1,21_ = 0.1845, *p* = 0.6719, treatment effect: *F*_1,21_ = 0.07035, *p* = 0.7934, two-way ANOVA; *n* = 6-7 per group). (e) Mifepristone effect on GR occurred in the HC regardless of the diet condition (treatment effect: *F*_1,17_ = 5.991, *p* < 0.05, two-way ANOVA; *n* = 4-5 per group). (f) A significant interaction between diet and treatment was revealed on FKBP51 expression in the hippocampus (interaction: *F*_1,16_ = 4.631, *p* < 0.05, two-way ANOVA, Bonferroni's test; *n* = 4 per group). (g) The expression of E6AP in the HC of n-3 deficient mice was reduced as compared to control diet mice whatever the treatment (interaction: *F*_1,20_ = 0.3197, *p* = 0.5781, diet effect: *F*_1,20_ = 4.571, *p* = 0.0451, treatment effect: *F*_1,20_ = 1.250, *p* = 0.2769, two-way ANOVA; *n* = 5–7 per group). A significant interaction between diet and treatment was revealed on both (h) social interaction (interaction: *F*_1,31_ = 16.27, *p* < 0.001, two-way ANOVA, Bonferroni's test; *n* = 8–10 per group) and (i) open-field test (interaction: *F*_1,38_ = 7.309, *p* < 0.05, two-way ANOVA, Bonferroni's test; *n* = 10-11 per group). Data are displayed as mean ± SEM. ^#^ *p* < 0.09; ^*∗*^ *p* < 0.05; ^*∗∗*^ *p* < 0.01; ^*∗∗∗*^ *p* < 0.001.

In patients with major depression, diminished GR expression or function has been postulated as causative factor for their increased HPA axis activity. We then asked whether altered GR signaling pathway in distinct brain structures positioned to inhibit the glucocorticoids stress response could partially explain HPA axis hyperactivity observed in n-3 deficient mice. Indeed, GR-mediated signaling in PFC and hippocampus is a pivotal component that contributes to the stress response, particularly in the context of HPA axis feedback desensitisation. In order to evaluate GR signaling in those structures, we used a Western blot method and analysed GR protein as well as FK506 binding protein 51 (FKBP51) protein expression which contains a glucocorticoid response element (GRE) and is modulated by glucocorticoids [24]. We found that expressions of GR and GR-responsive gene FKBP51 were downregulated in PFC of n-3 deficient mice as compared to control diet mice (Figures 3(b) and 3(c)). This result suggests that GR signaling pathway is inactivated in the PFC of n-3 deficient mice. If this hypothesis is true, the effect of chronic GR inactivation with the GR antagonist mifepristone on GR signaling in the PFC should not be detected in n-3 deficient mice. Consistent with this scenario, we found that mifepristone in n-3 deficient mice did not induce any further alterations on both GR (Figure 3(b)) and FKBP51 (Figure 3(c)) expressions in the PFC. Also, GR occlusion experiment revealed that GR signaling impairment found in the PFC of n-3 deficient mice was similar to that observed in control diet mice treated with mifepristone. Although FKBP51 expression was significantly higher in the hippocampus of n-3 deficient mice as compared to control diet mice (Figure 3(f)), no change in GR expression was found in the hippocampus (Figure 3(e)). In addition, we found that expression of GR in the hippocampus was reduced after 21 days of mifepristone treatment whatever the diet group (Figure 3(e)), revealing intact GR response to mifepristone in the hippocampus of n-3 deficient mice. Finally, we found that expression of the transcriptional coactivator of GRs E6-associated protein (E6AP) was unaffected by the diet or the treatment in the PFC of n-3 deficient mice as compared to the control diet mice (Figure 3(d)). On the contrary, the hippocampus of n-3 deficient mice showed a significant increase of the expression of E6AP whatever the treatment as compared to the control diet mice (Figure 3(g)).

At the emotional level, exploratory behaviours with emotional load (open-field and social interaction) are impaired in mice with defective HPA axis function [25–27]. We found that n-3 deficient mice did not engage new mice socially and exhibited anxiety-like behaviour in an open-field test, consistent with previous reports from our group [1, 19, 20]. This was revealed by a reduction in the number of active explorations of a new unfamiliar mouse (Figure 3(h)) and in the time spent exploring the centre of an open field (Figure 3(i)) compared to control diet mice. Interestingly, n-3 deficient mice displayed a behavioural impairment comparable with control diet mice treated with the GR antagonist mifepristone. Increased anxiety-related and social avoidance behaviour induced by mifepristone treatment was not linked to impaired motor locomotion. We found no difference regarding the total distance travelled whatever the group (data not shown). Altogether, these findings suggest that altered GR signaling pathway in PFC is closely associated with social and anxiety-related behaviours deficits induced by nutritional n-3 PUFAs deficiency.

**Figure 4:**
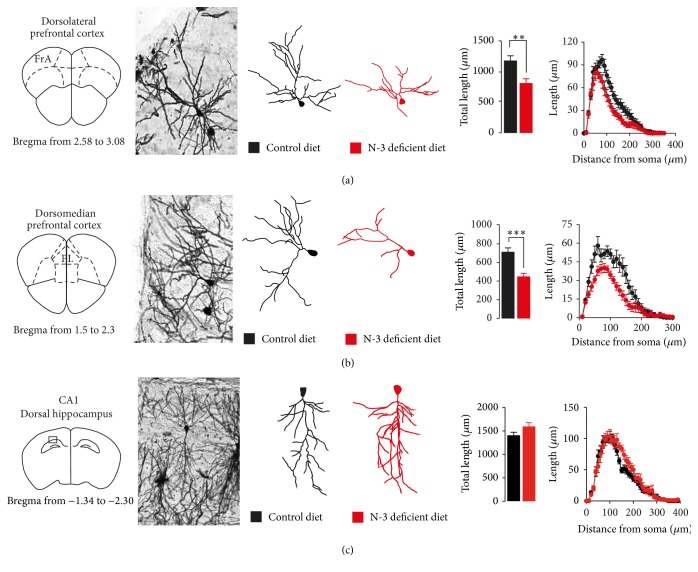
Effect of maternal n-3 deficient diet on dendritic arborisation in the PFC and hippocampus. (a, b, c left) dlPFC, dmPFC, and dorsal CA1 regions are represented. N-3 deficient diet induced shrinking of apical dendritic arborisation on pyramidal neurons of dlPFC (*t*_28_ = 3.323, ^*∗∗*^ *p* < 0.01, unpaired *t*-test, *n* = 15 neurons per group) and of dmPFC (*t*_34_ = 4.125, ^*∗∗∗*^ *p* < 0.001, unpaired *t*-test, *n* = 16 neurons per group). Dietary n-3 PUFAs deficient diet did not alter dendritic arborisation in the dorsal CA1 of the hippocampus (*t*_25_ = 1.791, *p* > 0.05, unpaired *t*-test, *n* = 14 neurons per group). Data are displayed as mean ± SEM.

Glucocorticoid receptor signaling has a crucial role in shaping neuronal morphology [28]. We have previously shown that nutritional n-3 PUFAs deficiency results in neuronal atrophy in the PFC [19]. Here, we aimed at investigating neuronal morphology in an additional structure, the hippocampus, in which no change was observed in GR protein levels in n-3 deficient mice. 3D morphometric analysis of Golgi-impregnated neurons revealed that exposure to nutritional n-3 PUFAs deficiency induced detrimental effects on the PFC pyramidal neurons. This was shown by a decrease in the total apical dendritic material in both dlPFC and dmPFC (Figures 4(a) and 4(b)). Furthermore, Sholl analysis was employed to determine the region of the apical dendritic tree at which the reduction in dendritic material was seen [29, 30]. This analysis revealed that, relative to control diet mice, dietary n-3 PUFAs deficiency reduced dendritic material in dlPFC and dmPFC at segments which were 80–120 *μ*m and 80–150 *μ*m from the soma, respectively. Interestingly, no change in total apical dendritic material was found in the dorsal CA1 of hippocampal pyramidal neurons of n-3 deficient mice as compared to control diet mice (Figure 4(c)). No significant changes were observed in Sholl analysis for apical dendritic arborisation in the dorsal CA1 between control diet and n-3 deficient mice in total length. Collectively, these data suggest region-specific alteration of PFC pyramidal neurons morphology and function.

## 4. Discussion

The brain is highly enriched in long-chain polyunsaturated fatty acids including arachidonic acid (AA) and docosahexaenoic acid (DHA) that regulate both the structure and the function of neurons [18]. Hence, it is not surprising that modification of brain PUFAs contents might be involved in the genesis of neuropsychopathologies. Nutritional n-3 PUFAs deficiency observed in Western countries has been associated with many diseases including anxiety and depression but the underlying pathophysiological mechanisms remain poorly understood. Here, we report that mice fed with a deficient diet in n-3 PUFAs demonstrated the ability to inhibit HPA axis after dexamethasone treatment which seems to be inconsistent with a depression-like phenotype. This suggests functional negative feedback which may be partially attributable to normal GR expression observed in the hippocampus of n-3 deficient mice. Indeed, the hippocampus is known to be strongly involved in GR-mediated negative feedback to the paraventricular nucleus of the hypothalamus [31]. To support this idea, we found that mifepristone reduced GR expression in the hippocampus regardless of the diet condition suggesting intact GR functionality in the hippocampus of n-3 deficient mice. In addition, acting as an inhibitor of GR activity, FKBP51 determines GR binding affinity to glucocorticoids. Its expression is also activated by glucocorticoids and is involved in an intracellular ultrashort negative feedback loop for GR activity (see [32] for review). The present profile of high basal corticosterone levels and the overexpression of FKBP51 in the HC of n-3 deficient mice both support the hypothesis of an enhanced inhibition of corticosterone binding to the GR. Furthermore, the overexpression of the transcriptional coactivator of GR E6AP observed in the HC of n-3 deficient mice may explain the fact that no change in GR expression was found. Taken together, these findings suggest a possible compensatory phenomenon involving FKBP51 and E6AP that preserves GR integrity within the HC of n-3 deficient mice. In contrast to the HC, occlusion experiment with mifepristone altered GR signaling in the PFC of control mice, with no further alterations in n-3 deficient mice. Thus, nutritional n-3 PUFAs deficiency alone is able to occlude GR signaling, reflected by a downregulation of GR and GR-responsive gene FKBP51 expressions in the PFC. GR downregulation is unlikely due to transcriptional alterations as no change was found in the expression of the transcriptional coactivator E6AP in the PFC of control diet and n-3 deficient mice.

Interestingly, the disruption of GR-signaling observed in n-3 deficient mice was associated with social deficit and anxiety-like behaviour. Our data agree with previous findings showing that transgenic mice with decreased GR expression in the brain (50%) or GR coactivator Ube3a knockout mice display anxiety- and depression-like behaviours with a reduced expression of FKBP51 in the cortex [22, 25]. In addition, a recent study in a clinical cohort has demonstrated a link between FKBP51 and stress-related psychiatric disorders [33].

Finally, our data revealed distinct regional alterations of neuronal morphology after exposure to dietary n-3 PUFAs deficiency. Nutritional n-3 PUFAs deficiency results in a decrease in the total apical length in the PFC, while no change was observed in the dorsal CA1 region of the hippocampus. In addition, Sholl analysis revealed that this atrophy in dendritic arborisation was driven by a loss of dendritic material restricted to proximal/middle regions of the apical dendritic arborisation (i.e., regions that were short of 180 *μ*m from the cell body). Proximal/middle apical dendrites project radially to local pyramidal cells and interneurons and are the primary target of intracortical projections, while distal apical dendrites in layer II/III pyramidal PFC neurons receive projection from both hippocampal areas CA1 and CA3. Thus, the atrophy of proximal/middle dendrites of layer II-III pyramidal neurons observed within the PFC might be a result of loss of intracortical input with intact input from hippocampal pyramidal neurons. At the behavioural level, both chronic inactivation of GR and nutritional n-3 PUFAs deficiency produced exploratory behaviour impairments as assessed by the open-field and the social interaction tests. As no change in GR expression and dendritic arborisation was found in hippocampal brain structure, the present study strongly suggests that GR sensitivity is closely associated with neuronal atrophy in the PFC of n-3 deficient mice that could be involved in social interaction deficits and increased anxiety-related behaviour. In conclusion, we here provide strong validity of the maternal n-3 PUFAs deficient diet as one of the many faces of stress that deeply affects GR-dependent HPA axis function and neuronal morphology plasticity in brain areas associated with emotional behaviour. This study reinforces the idea of the usefulness of the dietary n-3 PUFAs as an interesting tool for the design and testing of new nonpharmacological strategies in the treatment of neuropsychiatric disorders such as mood-related behaviours [19] and fragile X syndrome [34].

## Limitations

It is important to note some limitations of this work. First, corticosterone levels were analysed only at one time point. However, we are aware that it is necessary to take into account the strong circadian secretion rhythms involving glucocorticoids. Second, corticosterone experiments provide us with information regarding the total corticosterone secretion, including the free and protein-bound forms. In future studies, the measurement of both forms of corticosterone would help identify specific alteration induced by dietary n-3 PUFAs deficiency.

## References

[1] Lafourcade M., Larrieu T., Mato S. (2011). Nutritional omega-3 deficiency abolishes endocannabinoid-mediated neuronal functions. *Nature Neuroscience*.

[2] Maccari S., Morley-Fletcher S. (2007). Effects of prenatal restraint stress on the hypothalamus-pituitary-adrenal axis and related behavioural and neurobiological alterations. *Psychoneuroendocrinology*.

[3] Cao D., Kevala K., Kim J. (2009). Docosahexaenoic acid promotes hippocampal neuronal development and synaptic function. *Journal of Neurochemistry*.

[4] Hibbeln J. R. (1998). Fish consumption and major depression. *The Lancet*.

[5] Simopoulos A. P. (2002). The importance of the ratio of omega-6/omega-3 essential fatty acids. *Biomedicine & Pharmacotherapy*.

[6] Simopoulos A. P. (2009). Evolutionary aspects of the dietary omega-6:omega-3 fatty acid ratio: medical implications. *World Review of Nutrition and Dietetics*.

[7] Adams P. B., Lawson S., Sanigorski A., Sinclair A. J. (1996). Arachidonic acid to eicosapentaenoic acid ratio in blood correlates positively with clinical symptoms of depression. *Lipids*.

[8] Green P., Hermesh H., Monselise A., Marom S., Presburger G., Weizman A. (2006). Red cell membrane omega-3 fatty acids are decreased in nondepressed patients with social anxiety disorder. *European Neuropsychopharmacology*.

[9] McNamara R. K., Liu Y. (2011). Reduced expression of fatty acid biosynthesis genes in the prefrontal cortex of patients with major depressive disorder. *Journal of Affective Disorders*.

[10] Bondi C. O., Taha A. Y., Tock J. L. (2014). Adolescent behavior and dopamine availability are uniquely sensitive to dietary omega-3 fatty acid deficiency. *Biological Psychiatry*.

[11] Takeuchi T., Iwanaga M., Harada E. (2003). Possible regulatory mechanism of DHA-induced anti-stress reaction in rats. *Brain Research*.

[12] DeMar J. C. Jr., Ma K., Bell J. M., Igarashi M., Greenstein D., Rapoport S. I. (2006). One generation of n-3 polyunsaturated fatty acid deprivation increases depression and aggression test scores in rats. *The Journal of Lipid Research*.

[13] Chrousos G. P. (2009). Stress and disorders of the stress system. *Nature Reviews Endocrinology*.

[14] de Kloet E. R. (2014). From receptor balance to rational glucocorticoid therapy. *Endocrinology*.

[15] Nemeroff C. B., Widerlov E., Bissette G. (1984). Elevated concentrations of CSF corticotropin-releasing factor-like immunoreactivity in depressed patients. *Science*.

[16] Niwa M., Jaaro-Peled H., Tankou S. (2013). Adolescent stress-induced epigenetic control of dopaminergic neurons via glucocorticoids. *Science*.

[17] Holsboer F. (2000). The corticosteroid receptor hypothesis of depression. *Neuropsychopharmacology*.

[18] Bazinet R. P., Layé S. (2014). Polyunsaturated fatty acids and their metabolites in brain function and disease. *Nature Reviews Neuroscience*.

[19] Larrieu T., Hilal L. M., Fourrier C. (2014). Nutritional omega-3 modulates neuronal morphology in the prefrontal cortex along with depression-related behaviour through corticosterone secretion. *Translational Psychiatry*.

[20] Larrieu T., Madore C., Joffre C., Layé S. (2012). Nutritional n-3 polyunsaturated fatty acids deficiency alters cannabinoid receptor signaling pathway in the brain and associated anxiety-like behavior in mice. *Journal of Physiology and Biochemistry*.

[21] Mingam R., Moranis A., Bluthé R.-M. (2008). Uncoupling of interleukin-6 from its signalling pathway by dietary n-3-polyunsaturated fatty acid deprivation alters sickness behaviour in mice. *European Journal of Neuroscience*.

[22] Ridder S., Chourbaji S., Hellweg R. (2005). Mice with genetically altered glucocorticoid receptor expression show altered sensitivity for stress-induced depressive reactions. *The Journal of Neuroscience*.

[23] Anacker C., Zunszain P. A., Carvalho L. A., Pariante C. M. (2011). The glucocorticoid receptor: pivot of depression and of antidepressant treatment?. *Psychoneuroendocrinology*.

[24] Lee R. S., Tamashiro K. L. K., Yang X. (2011). A measure of glucocorticoid load provided by DNA methylation of Fkbp5 in mice. *Psychopharmacology*.

[25] Godavarthi S. K., Dey P., Maheshwari M., Jana N. R. (2012). Defective glucocorticoid hormone receptor signaling leads to increased stress and anxiety in a mouse model of Angelman syndrome. *Human Molecular Genetics*.

[26] Barik J., Marti F., Morel C. (2013). Chronic stress triggers social aversion via glucocorticoid receptor in dopaminoceptive neurons. *Science*.

[27] Lehmann M. L., Brachman R. A., Martinowich K., Schloesser R. J., Herkenham M. (2013). Glucocorticoids orchestrate divergent effects on mood through adult neurogenesis. *The Journal of Neuroscience*.

[28] Suri D., Vaidya V. A. (2013). Glucocorticoid regulation of brain-derived neurotrophic factor: relevance to hippocampal structural and functional plasticity. *Neuroscience*.

[29] Hill M. N., Hillard C. J., McEwen B. S. (2011). Alterations in corticolimbic dendritic morphology and emotional behavior in cannabinoid CB_1_ receptor-deficient mice parallel the effects of chronic stress. *Cerebral Cortex*.

[30] Hill M. N., Kumar S. A., Filipski S. B. (2013). Disruption of fatty acid amide hydrolase activity prevents the effects of chronic stress on anxiety and amygdalar microstructure. *Molecular Psychiatry*.

[31] Herman J. P., Ostrander M. M., Mueller N. K., Figueiredo H. (2005). Limbic system mechanisms of stress regulation: hypothalamo-pituitary-adrenocortical axis. *Progress in Neuro-Psychopharmacology and Biological Psychiatry*.

[32] Binder E. B. (2009). The role of FKBP5, a co-chaperone of the glucocorticoid receptor in the pathogenesis and therapy of affective and anxiety disorders. *Psychoneuroendocrinology*.

[33] Fani N., Gutman D., Tone E. B. (2013). *FKBP5* and attention bias for threat: associations with hippocampal function and shape. *JAMA Psychiatry*.

[34] Pietropaolo S., Goubran M. G., Joffre C. (2014). Dietary supplementation of omega-3 fatty acids rescues fragile X phenotypes in Fmr1-Ko mice. *Psychoneuroendocrinology*.

